# ﻿Comparison of complete mitochondrial genome sequences in the *Aporrectodeacaliginosa* species group (Annelida, Crassiclitellata, Lumbricidae)

**DOI:** 10.3897/zookeys.1231.144623

**Published:** 2025-03-13

**Authors:** Csaba Csuzdi, Jachoon Koo, Yong Hong

**Affiliations:** 1 Kenderesi str. 39, Piliscsaba, Hungary; 2 Division of Science Education and Institute of Fusion Science, College of Education, Jeonbuk National University, Jeonju 54896, Republic of Korea; 3 Department of Agricultural Biology, College of Agriculture & Life Science, Jeonbuk National University, Jeonju 54896, Republic of Korea

**Keywords:** *Aporrectodeacaliginosa*, *Aporrectodeatrapezoides*, Lumbricidae, mitochondrial genome, phylogeny

## Abstract

We present for the first time the complete mitochondrial genomes (mt genomes) of the earthworms *Aporrectodeacaliginosa* and *Ap.trapezoides* (Clitellata, Megadrili) collected in Hungary and Korea, respectively.

The complete mt genome of *Ap.trapezoides* comprised 15,014 base pairs. Lengths of the three complete *Ap.caliginosa* mt genomes varied between 15,090 and 15,123 bp. All four mt genomes contained 13 protein-coding genes (PCGs), two rRNA genes, 22 tRNA genes, and one major non-coding control region. These mt genome arrangements are identical to those observed in the mt genomes of most earthworms, and all the 37 genes are transcribed from the same directional strand. All 13 PCGs had the same ATG start codon. Most of the PCGs end with TAA or TAG, whereas the remaining end with an incomplete stop codon, T. Stop codons were consistent in the PCGs throughout the mt genomes, except *Ap.caliginosa* 5, which contains a TAG stop codon in ND5 instead of the TAA found in the other samples. Both species’ genomes showed biased base composition, with 63.5% AT and 36.4% GC content in *Ap.trapezoides* and 62.8% and 37.2% in *Ap.caliginosa*. Phylogenetic analysis of the mt genomes corroborated the monophyly of the family Lumbricidae and the close relationship between *Ap.trapezoides* and *Ap.caliginosa* species pairs. The available *Ap.tuberculata* sequences were embedded between the *Ap.caliginosa* samples, thereby supporting the synonymy of the two names.

## ﻿Introduction

*Aporrectodeacaliginosa* was described as *Enterioncaliginosum* Savigny, 1826 and placed in Savigny’s "*Tribu*" No. 1 (Savigny 1826: 179). The "*Tribu*" No. 1 is defined as follows: “Les soies sont rapprochées par paires. La ceinture a de chaque côté deux pores qui correspondent chacun à un seul segment, et qui, si l’on compte celui qui les sépare, comprennent les trois pénultièmes” [The setae paired. The girdle (clitellum) has two pores (tubercles) on each side, each of which corresponds to a single segment, and which, if the one between them is counted, comprise the three penultimate segments]. The species *caliginosum* was defined as “La ceinture, de huit segmens, finit avec le trente-quatriéme du corps” [The clitellum, of eight segments, ending with the thirty-fourth of the body]. The diagnosis of *E.caliginosum* in modern terms according to Cain (1955: 482–483) is as follows: “Male pores with conspicuous lips, apertures on segment 15; setae paired; clitellum on 27–34; tubercula pubertatis on 31 and 33, two pairs of spermathecae opening on the ventral surface; four pairs of seminal vesicles; coelomic fluid not colored.”

The identity of *Enterioncaliginosum* has provoked a long and continuing debate (Blakemore 2008). Gates (1972a, 1972b) and other North American authors (e.g. Reynolds 1977, 2022) regarded Savigny’s definition as inadequate and used the junior synonym name *Allolobophoraturgida* Eisen, 1873 because Tétry (1937) erroneously cited the position of the tubercles as being on segment 31, 32 when revising Savigny’s material housed in the Muséum national d’Histoire naturelle in Paris. However, Tétry (1937) did not find the type material of *E.caliginosum* and Savigny (1826) never wrote of this (Cain 1955). As *E.caliginosum* is published according to ICZN Article 11 and its diagnosis satisfies Article 13, it is an available and valid senior name for this taxon.

Later, several synonyms (e.g. *Lumbricuslividus* Templeton, 1836; *Allolobophorasimilis* Friend, 1910 (see Blakemore 2008: 526 for a complete synonym list) and closely related taxa were described, such as *Lumbricustrapezoides* Dugés, 1828; *Allolobophoranocturna* Evans, 1946; Allolobophoraturgidavar.tuberculata (Eisen 1874). In addition, the latter three taxa, which together with *Ap.caliginosa*, form the *Ap.caliginosa* species group (Blakemore 2002), have been treated differently by different authors. For instance, Pop (1949), Zicsi (1982), and Csuzdi and Zicsi (2003) regarded all three species names as synonyms of *Ap.caliginosa*. Gates (1972a) and Reynolds (1977, 2022) considered *Ap.trapezoides*, *Ap.tuberculata*, and *Ap.nocturna* independent taxa from *Ap.caliginosa* (syn. *Ap.turgida*). Mršić (1991) considered *Ap.tuberculata* as a synonym for *Ap.caliginosa*, *Ap.trapezoides* as a subspecies of *Ap.caliginosa*, and *Ap.nocturna* as a valid species. Briones (1996) reviewed the *Ap.caliginosa* species group and concluded that there were only two valid taxa, *Ap.caliginosa* and *Ap.trapezoides*. However, the author did not comment on the status of *Ap.nocturna* owing to a lack of sufficient material.

To further complicate this issue, Bouché (1972) distinguished three subspecies within *Ap.caliginosa*, along with several invalid infrasubspecific taxa (varieties), that he placed in his genus *Nicodrilus* Bouché, 1972, a junior synonym of *Aporrectodea* Örley, 1885 (Sims and Gerard 1985).

The *Ap.caliginosa* species group is one of the most molecularly studied earthworm taxa. Early multigene phylogenetic analyses (Pérez-Losada et al. 2009; Fernández et al. 2011, 2012, 2013; Briard et al. 2012) revealed high genetic variability in the species group and the presence of several highly divergent cryptic lineages in different constituent taxa. *Aporrectodeatuberculata* with *Ap.caliginosa* form a well-separated clade from the other taxa in this complex. However, *Ap.caliginosa* was paraphyletic without *Ap.tuberculata*, and *Ap.trapezoides* comprised two highly separated clades and appeared to be polyphyletic, as clade I is more closely related to the clade comprising *Ap.nocturna*, *Ap.giardi* (valid name *Ap.terrestris*), and *Ap.longa*. Later studies either corroborated these results (Shekhovtsov et al. 2017; Marchán et al. 2020) or found *Ap.caliginosa* polyphyletic (Shekhovtsov et al. 2016; Latif et al. 2020).

Over the past few years increasing number of complete mitochondrial genomes (mt genomes) have been published, facilitating the differentiation of closely related species (Zhang et al. 2016; Shekhovtsov et al. 2020; Csuzdi et al. 2022). Recently, Zhao et al. (2022) reported several nearly complete lumbricid mt genomes, including one *Ap.trapezoides* and six *Ap.tuberculata* sequences. Interestingly, one of the reported *Ap.tuberculata* sequences is registered as *Ap.caliginosa* in the GenBank (sample 420Ra, accession number NC_066400).

Herein, we report the complete mt genomes of the Korean *Ap.trapezoides* and three Hungarian *Ap.caliginosa* specimens, as well as compare these data to the published complete or nearly complete mt genomes of the *Ap.caliginosa* species group to help solve this continuing taxonomic issue.

## ﻿Material and methods

### ﻿Sample preparation and DNA extraction

Adult *Ap.trapezoides* were collected from a farm in Seongsu-myeon, Imsil-gun, Jeollabuk-do, Korea (33°41'23.80"N, 126°38'33.67"E; 40 m a.s.l.) on March 28, 2021 and preserved in 99% ethanol until DNA extraction. *Ap.caliginosa* specimens were collected in a deciduous forest patch near Szendehely, Hungary (47°52'32.7"N, 19°6'16.6"E) on May 4, 2021, and preserved in 96% ethanol until DNA extraction. Voucher specimens of each species were deposited at Jeonbuk National University, Jeonju City, Korea. Total genomic DNA was prepared from a small portion of the body segments of a single adult earthworm using a QIAamp DNA Mini Kit (Qiagen, Hilden, Germany). The remaining tissue was preserved at −20 °C in 90% ethanol.

### ﻿TruSeq DNA library construction

The sequencing library was prepared by random fragmentation of genomic DNA, followed by 5’ and 3’ adapter ligations using the Illumina TruSeq DNA Nano Library Prep Kit according to the manufacturer’s instructions (Illumina Inc., San Diego, CA, USA). The resulting libraries were quantified through a qPCR-based assay using the KAPA Library Quantification Kit for Illumina sequencing platforms, according to the manufacturer’s instructions (Kapa Biosystems, Woburn, MA, USA). Libraries were qualified using the Agilent Technologies 2200 TapeStation (Agilent Technologies, Santa Clara, CA, USA).

### ﻿DNA sequencing, assembly, and annotation

Paired-end (2×150 base pairs [bp]) sequencing was performed using the Illumina HiSeq-X platform (Illumina Inc.) at Macrogen Inc. (Seoul, Korea). To reduce bias in the analysis, adapter trimming and quality filtering were performed using Trimmomatic v. 0.36 (Bolger et al. 2014). *De novo* assembly of raw sequencing reads was performed using various *k*-mer lengths in SPAdes v. 3.13.0 (Bankevich et al. 2012). Mitochondrial contigs were assembled into a single contig using BLASTN alignment (https://blast.ncbi.nlm.nih.gov/Blast.cgi) against the *Lumbricusterrestris* mitogenome (GenBank accession number NC_001673) as the reference sequence. Annotation and visualization of mt genomes were performed using the online MITOS software (Donath et al. 2019), and manual curation was performed using BLAST searches in the NCBI database for various earthworm mt genomes (Table 1). A comparative map of the mt genomes was created using Geneious Prime 2024 software. The *cox1* sequence was used as an anchor for the linearized mt genome maps. The annotated complete genome sequences were registered in GenBank under the accession numbers PQ572750–PQ572753.

**Table 1.** List of Megadrili mitogenomes used in this study.

Family	Taxa	Genbank No.	Total length (bp)	Reference
Megascolecidae	*Amynthasaspergillus*	KJ830749	15,115	Zhang et al. 2016
	*Amynthascarnosus*	KT429008	15,160	Zhang et al. 2016
	*Amynthascorticis*	KM199290	15,126	Zhang et al. 2015
	*Amynthascucullatus*	KT429012	15,122	Zhang et al. 2016
	*Amynthasgracilis*	KP688582	15,161	Zhang et al. 2015
	*Amynthashupeiensis*	KT429009	15,069	Zhang et al. 2016
	*Amynthasjiriensis*	KT783537	15,151	Hong et al. 2017
	*Amynthaslongisiphonus*	KM199289	15,176	Zhang et al. 2015
	*Amynthasmoniliatus*	KT429020	15,133	Zhang et al. 2016
	*Amynthasmorrisi*	KT429011	15,026	Zhang et al. 2016
	*Amynthaspectiniferus*	KT429018	15,188	Zhang et al. 2016
	*Amynthasrobustus*	KT429019	15,013	Zhang et al. 2016
	*Amynthasseungpanensis*	OL321943	15,085	Kim and Hong 2022
	*Amynthas* sp. 1	KT429010	15,131	unpublished
	*Amynthas* sp. 2 JS-2012	KT429007	15,159	unpublished
	*Amynthas* sp. 2	KT429014	15,086	unpublished
	*Amynthas* sp. 3	KT429013	15,152	unpublished
	*Amynthastriastriatus*	KT429016	15,160	Zhang et al. 2016
	*Amynthasyunoshimensis*	LC573969	15,109	Seto et al. 2021
	*Duplodicodrilusschmardae*	KT429015	15,156	Zhang et al. 2016
	*Metaphirecalifornica*	KP688581	15,147	Zhang et al. 2015
	*Metaphireguillelmi*	KT429017	15,174	Zhang et al. 2016
	*Metaphirehilgendorfi*	LC573968	15,186	Seto et al. 2021
	*Metaphirevulgaris*	KJ137279	15,061	Zhang et al. 2014
	*Perionyxexcavatus*	EF494507	15,083	unpublished
	*Tonoscolexbirmanicus*	KF425518	15,170	Wang et al. 2015
Lumbricidae	*Aporrectodearosea*	MK573632	15,086	Shekhovtsov and Peltek 2019
	*Aporrectodeacaliginosa*	**PQ572750**	**15,090**	**this study**
		**PQ572751**	**15111**	**this study**
		**PQ572752**	**15123**	**this study**
		CM035405	15,120	unpublished
		NC_066400	15,089	Zhao et al. 2022
	*Aporrectodeatrapezoides*	**PQ572753**	**15,014**	**this study**
		OM687882	14,998	Zhao et al. 2022
	*Aporrectodeatuberculata*	OL840317	15,058	Zhao et al. 2022
		OM687883	15,125	Zhao et al. 2022
		OM687884	15,126	Zhao et al. 2022
		OM687885	15,129	Zhao et al. 2022
		OM687886	15,116	Zhao et al. 2022
	*Bimastusparvus*	MZ857199	15,194	Liu et al. 2021
	*Dendrobaenaoctaedra*	MZ857197	15715	Liu et al. 2021
	*Eiseniaandrei*	OK513069	15,714	Csuzdi et al. 2022
	*Eiseniafetida*	OK513070	16,560	Csuzdi et al. 2022
	*Eisenianordenskioldi*	MZ857200	15152	Liu et al. 2021
		OM687887	16,114	Zhao et al. 2022
		OL840314	15,290	Zhao et al. 2022
	*Lumbricusrubellus*	MN102127	15,464	Zhang et al. 2019
	*Lumbricusterrestris*	U24570	14,998	Boore and Brown 1995
	*Octolasiontyrtaeum*	MZ857201	14,977	Liu et al. 2021
Moniligastridae	*Drawidajaponica*	KM199288	14,648	Zhang et al. 2016
Rhinodrilidae	*Pontoscolexcorethrurus*	KT988053	14,835	Conrado et al. 2017

### ﻿Phylogenetic analyses

To check the identity of the morphologically identified specimens, the *cox1* barcoding regions were analyzed with barcodes selected and downloaded from the BOLD v. 4 database (Ratnasingham and Hebert 2013) using the IQ-tree web server with default settings and suggested TPM2u+F+I+G4 substitution model.

To clarify the phylogenetic positions of the two taxa, complete or nearly complete mitogenomes were obtained from GenBank, comprising 26 species of Megascolecidae and 12 species of Lumbricidae. *Pontoscolexcorethrurus* (Müller, 1857) from the Rhinodrilidae family and *Drawidajaponica* (Michaelsen, 1892) from the exquisiclitellate family Moniligastridae were used as outgroups. Phylogenetic analysis was conducted based on two datasets, a complete set of 37 mitochondrial genes and subset comprising only 13 protein-coding genes (PCGs), using maximum likelihood (ML) and Bayesian (BI) methods. Sequences for each gene from the 40 earthworm species (Table 1) were individually aligned using MAFFT v. 7 (Katoh and Standley 2013) and trimmed using TrimAl v. 1.2 (Capella-Gutiérrez et al. 2009). The trimmed sequences were concatenated into a single multi-gene alignment. The best-fit substitution model for each dataset was selected using ModelFinder (Kalyaanamoorthy et al. 2017) integrated within the IQ-TREE (Nguyen et al. 2014) based on the Bayesian Information Criterion (Schwarz 1978). The ML phylogenetic trees were constructed using IQ-TREE for both datasets by applying the selected GTR+F+I+I+R5 model. In addition, a partitioned ML analysis was also performed on the 13 PCG dataset using the best-fit models TIM2+F+R7 for *atp6*+*atp8*+*nd2*+*nd6* genes, GTR+F+I+G4 for *cox1*+*cox2*+*cox3*+*cytb* genes and TIM2+F+R10 for *nd1*+*nd3*+*nd4*+*nd4L*+*nd5* genes. Branch support was assessed using 5,000 ultrafast bootstrap replicates (Hoang et al. 2018). Bayesian analysis was performed using MrBayes v. 3.2.7 (Ronquist et al. 2012) with two independent runs set to 10 million generations each and sampling every 1000^th^ generation (10,000 trees). Twenty-five percent of the trees were discarded as burn-in, and the remaining trees were combined and summarized into a 50% majority-rule consensus tree.

Estimation of the evolutionary divergences between the analyzed *Ap.caliginosa* group sequences were conducted in MEGA X (Kumar et al. 2018) using the uncorrected *p*-distance. The analyzed matrix was 14,681 bp long and contained only coding regions.

## ﻿Results

**Figure 1.**
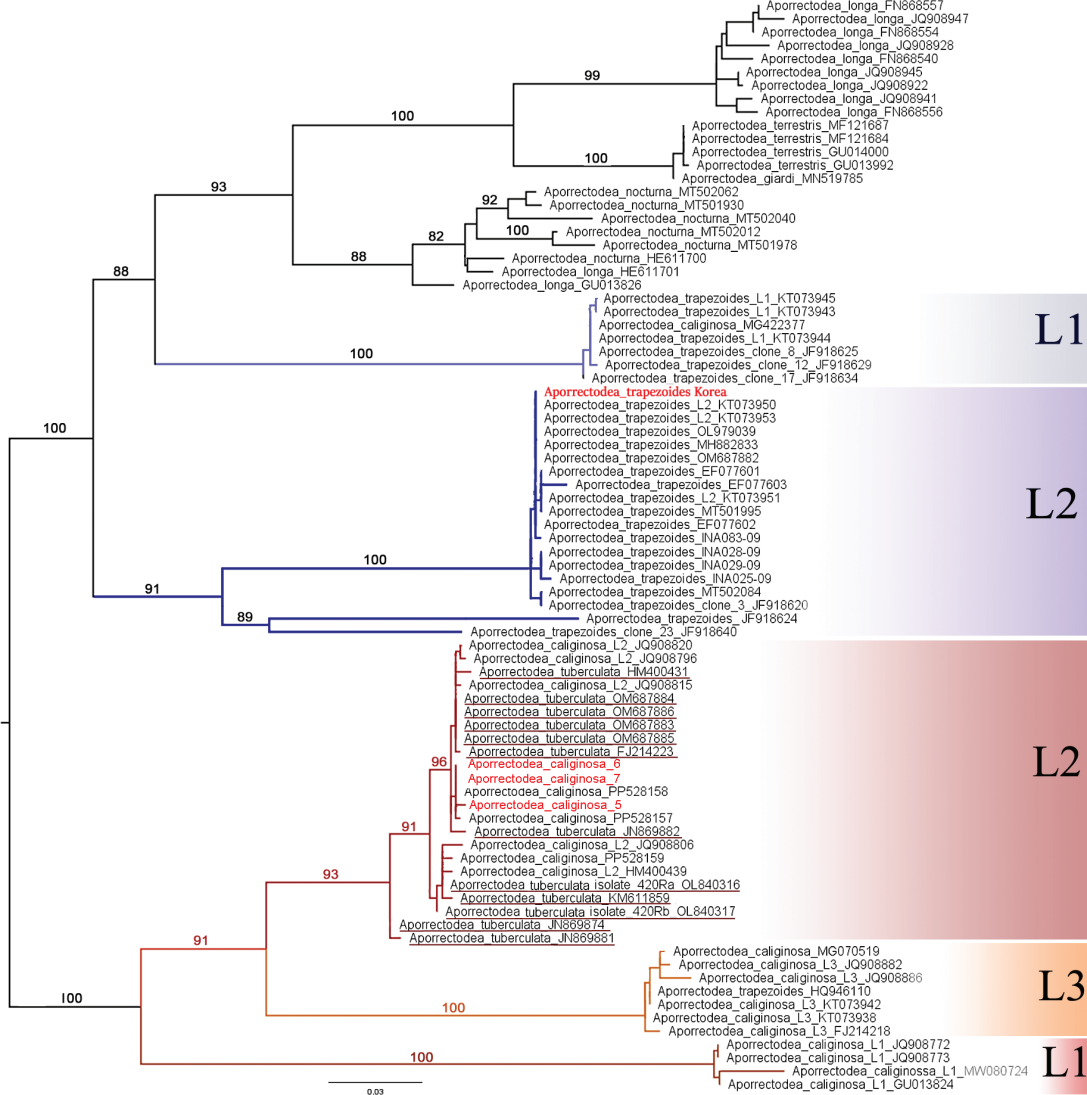
Maximum-likelihood (ML) tree based on the *cox1* gene fragments with bootstrap values of selected *Aporrectodeacaliginosa* group specimens. Red names represent new sequences. L1, L2, and L3 refer to different lineages. *Ap.tuberculata* sequences are underlined.

Based on the analysis of the barcoding region of *cox1* gene, our newly sequenced *Ap.trapezoides* specimen belongs to the widely distributed lineage 2 (Fernández et al. 2011). The *Ap.caliginosa* specimens analyzed were nested among the *caliginosa* lineage 2 specimens (Porco et al. 2013; Shekhovtsov et al. 2016), together with all *Ap.tuberculata* and *Ap.caliginosa* samples reported in the recent study by Zhao et al. (2022) (Fig. 1).

**Figure 2.**
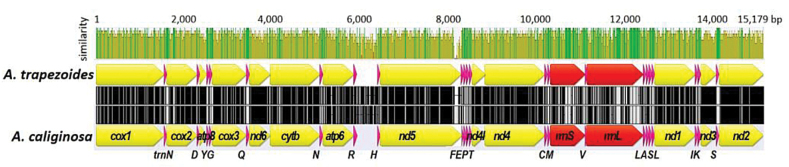
Comparison of the mitogenomes of *Aporrectodeatrapezoides* and *Ap.caliginosa* (sample no. 5). Map was constructed based on sequence similarity using Geneious Prime 2021 software. Sequence similarity is represented by green (100%), brown (30–99%), and red (<30%). Yellow, pink, and red arrows indicate PCGs, tRNAs, and rRNAs, respectively. Organization of mitochondrial genes is described in Table 2. Non-coding (nc) region is defined between *trnR* and *trnH*.

The complete mt genome of *Ap.trapezoides* comprises 15,014 bp. The length of the three *Ap.caliginosa* mt genomes analyzed in this study varied between 15,090 and 15,123 bp. The mitogenome setup of both species followed the typical bauplan of the earthworm mitogenome assembly, consisting of 13 PCGs, 22 transfer RNAs, two ribosomal RNA genes, and a control region (Fig. 2; Table 2).

**Table 2.** Comparative analysis of gene organization of *Aporrectodeatrapezoides* and *Ap.caliginosa* mitogenomes.

Gene	*Ap.caliginosa*		*Ap.caliginosa*		*Ap.tuberculata*		*Ap.trapezoides*		*Ap.trapezoides*		*Ap.caliginosa* 5		Similarity (%)*
	(CM035405)		(NC_066400)		(OL840317)		(OM687882)		(this study)		(this study)		
	Size (bp)	start/stop codon	Size (bp)	start/stop codon	Size (bp)	start/stop codon	Size (bp)	start/stop codon	Size (bp)	start/stop codon	Size (bp)	start/stop codon	
*cox1*	1540	ATG/T	1540	ATG/T	1540	ATG/T	1540	ATG/T	1540	ATG/T	1540	ATG/T	84.50%
*trnN*	61	–	61	–	61	–	61	–	61	–	61	–	88.9
*cox2*	687	ATG/TAG	687	ATG/TAG	687	ATG/TAG	687	ATG/TAG	687	ATG/TAG	687	ATG/TAG	85.9
*trnD*	61	–	61	–	61	–	61	–	61	–	61	–	90.2
*atp8*	160	ATG/T	160	ATG/T	160	ATG/T	160	ATG/T	160	ATG/T	160	ATG/T	71.2
*trnY*	63	–	63	–	63	–	62	–	62	–	63	–	95.2
*trnG*	63	–	63	–	63	–	63	–	63	–	63	–	93.7
*cox3*	778	ATG/T	778	ATG/T	778	ATG/T	778	ATG/T	778	ATG/T	778	ATG/T	83.8
*trnQ*	69	–	69	–	69	–	69	–	69	–	69	–	100
*nad6*	468	ATG/TAA	468	ATG/TAA	468	ATG/TAA	468	ATG/TAA	468	ATG/TAA	468	ATG/TAA	77.8
*cytb*	1140	ATG/TAA	1140	ATG/TAA	1140	ATG/TAA	1140	ATG/TAA	1140	ATG/TAA	1140	ATG/TAA	83
*trnW*	64	–	64	–	65	–	62	–	62	–	65	–	89.2
*atp6*	696	ATG/TAA	696	ATG/TAA	696	ATG/TAA	696	ATG/TAA	696	ATG/TAA	696	ATG/TAA	80.4
*trnR*	63	–	63	–	63	–	65	–	65	–	63	–	80.6
*^#^NC*	437	–	407	–	374	–	440	–	456	–	409	–	61
*trnH*	62	–	62	–	62	–	62	–	62	–	62	–	82.8
*nad5*	1722	ATG/TAA	1722	ATG/TAA	1722	ATG/TAA	1722	ATG/TAA	1722	ATG/TAA	1722	ATG/TAG	79.6
*trnF*	61	–	61	–	61	–	61	–	61	–	61	–	91.8
*trnE*	63	–	63	–	63	–	63	–	63	–	63	–	92.3
*trnP*	64	–	64	–	64	–	64	–	64	–	64	–	90.8
*trnT*	63	–	63	–	64	–	63	–	63	–	63	–	93.7
*nad4L*	297	ATG/TAA	297	ATG/TAA	297	ATG/TAA	297	ATG/TAA	297	ATG/TAA	297	ATG/TAA	79.9
*nad4*	1359	ATG/TAG	1359	ATG/TAG	1359	ATG/TAG	1350	ATG/TAG	1359	ATG/TAG	1359	ATG/TAG	80.8
*trnC*	65	–	65	–	65	–	65	–	65	–	65	–	90.9
*trnM*	63	–	63	–	63	–	63	–	63	–	63	–	96.8
*rrnS*	788	–	788	–	788	–	789	–	795	–	794	–	89
*trnV*	63	–	63	–	63	–	63	–	63	–	63	–	93.8
*rrnL*	1257	–	1256	–	1256	–	1256	–	1275	–	1295	–	86
*trnL*	62	–	62	–	62	–	62	–	62	–	62	–	93.7
*trnA*	62	–	62	–	62	–	62	–	62	–	62	–	88.7
*trnS*	67	–	67	–	67	–	67	–	67	–	67	–	94.1
*trnL*	62	–	62	–	62	–	62	–	62	–	62	–	98.4
*nad1*	922	ATG/T	922	ATG/T	922	ATG/T	922	ATG/T	922	ATG/T	922	ATG/T	83.6
*trnI*	66	–	66	–	66	–	64	–	64	–	66	–	90.9
*trnK*	63	–	63	–	63	–	64	–	64	–	63	–	92.3
*nad3*	354	ATG/TAG	354	ATG/TAG	354	ATG/TAG	354	ATG/TAG	354	ATG/TAG	354	ATG/TAG	74.6
*trnS*	64	–	64	–	64	–	64	–	64	–	64	–	100
*nad2*	1006	ATG/T	1006	ATG/T	1006	ATG/T	1006	ATG/T	1006	ATG/T	1006	ATG/T	79.1

^#^Putative non-coding region between *trnR* and *trnH*. * indicates the similarity of nucleotide sequence between *Ap.trapezoides* and *Ap.caliginosa* (Apc-5).

All genes were encoded on the heavy DNA strand, and both genomes showed biased base composition, with 63.5% AT and 36.4% GC content in *Ap.trapezoides* and 62.8% and 37.2% in *Ap.caliginosa*.

Overall mitogenome sequence similarity between the two species (*Ap.trapezoides* and *Ap.caliginosa* no. 5) was 80.8% and it increased to 85.8% when the control region was excluded. The 13 PCGs showed 71.2–85.9% similarity (Table 2). Among the PCGs, *cox2* showed the highest similarity (85.9%) and *atp8* the lowest (71.2%). The average similarity of the 13 PCGs between the two species was 84%. However, the deduced amino acid sequences of the 13 PCGs showed 92.7% similarity on average across species; *cox1* was the most similar (99.4%), and *atp8* the most dissimilar (66.04%; Table 3). The sequence variation between the two species was lower at the amino acid level than at the DNA level. In particular, *cox1* showed 82.47–84.41% similarity at the DNA level, but >99% similarity at the amino acid level (Table 4).

**Table 3.** Comparison of deduced amino acid composition of 13 PCGs in the *Aporrectodeacaliginosa* species group.

		*Ap.trapezoides* this study vs.	*Ap.trapezoides* this study vs.	*Ap.trapezoides* this study vs.	*Ap.caliginosa* (CM035405) vs.	*Ap.caliginosa* (Apc-5) vs.
Protein	aa size	*A.caliginosa* (CM035405)	*A.caliginosa* (NC_066400)	*A.caliginosa* (Apc-5)	*A.caliginosa* (Apc-5)	*A.tuberculata* (OM687883)
cox1	513	99.40%	99.40%	99.40%	100%	100%
cox2	228	97.20%	96.73%	96.73%	99.53%	100%
atp8	53	66.04%	66.04%	67.92%	86.79%	96.23%
cox3	259	96.00%	95.60%	95.60%	98.00%	99.60%
nad6	156	86.71%	88.59%	86.58%	95.10%	97.99%
cytb	379	97.35%	94.99%	95.78%	97.88%	99.74%
atp6	231	89.96%	89.52%	89.52%	95.20%	99.13%
nad5	573	91.94%	91.97%	91.97%	97.55%	99.30%
nad4l	98	97.50%	91.84%	92.86%	92.86%	100%
nad4	452	90.78%	91.57%	91.59%	97.00%	99.12%
nad1	307	93.20%	94.77%	95.11%	95.58%	99.67%
nad3	117	92.71%	88.79%	86.32%	94.79%	96.58%
nad2	335	89.55%	87.74%	88.42%	95.82%	99.04%

* All 13 mitochondrial proteins from mitogenome showed same size across the species.

**Table 4.** Comparison of the nucleotide sequences of the 13 PCGs between *Ap.trapezoides* and *Ap.caliginosa*.

	*Ap.trapezoides* this study vs.	*Ap.trapezoides* this study vs.	*Ap.trapezoides* this study vs.	*A.caliginosa* (CM035405) vs.	*A.caliginosa* 5 this study vs.
Protein	*A.caliginosa* (CM035405)	*A.caliginosa* (NC_066400)	*A.caliginosa* 5 this study	*A.caliginosa* 5 this study	*A.tuberculata* (OM687883)
cox1	82.47%	84.93%	84.41%	88.05%	99.03%
cox2	85.20%	85.96%	85.82%	89.88%	99.12%
atp8	74.36%	73.58%	72.33%	84.62%	98.74%
cox3	83.20%	84.04%	83.53%	88.53%	99.49%
nad6	79.87%	78.49%	77.63%	84.12%	98.49%
cytb	82.94%	83.03%	82.76%	86.74%	99.03%
atp6	80.06%	79.80%	80.23%	86.03%	98.70%
nad5	79.04%	79.76%	79.64%	87.10%	98.78%
nad4l	78.75%	79.93%	79.59%	82.50%	100%
nad4	91.56%	80.68%	80.68%	86.28%	98.67%
nad1	82.16%	84.69%	83.93%	86.73%	98.59%
nad3	77.78%	76.92%	76.07%	84.05%	97.72%
nad2	78.92%	78.81%	79.20%	85.64%	99.10%

**Figure 3.**
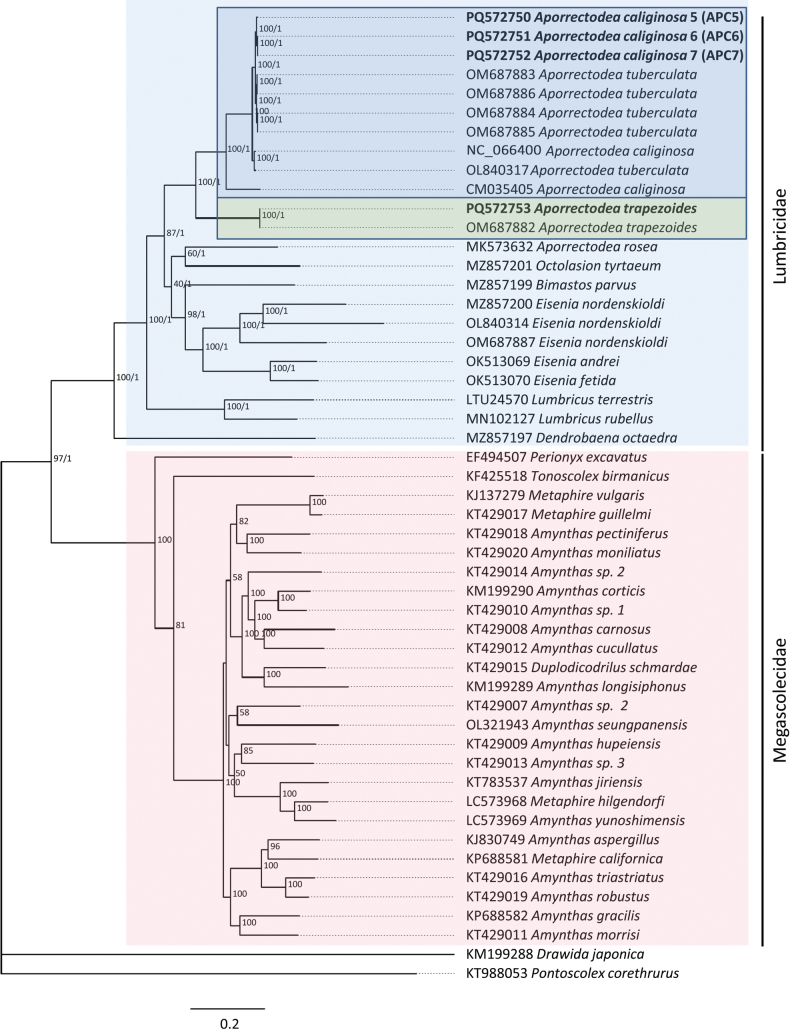
Phylogenetic analysis of 40 Megadrili group spp., including *Aporrectodeatrapezoides* and *Ap.caliginosa*, based on sequences of 37 mitochondrial genes. Numbers beside nodes are ML bootstrap values and BI posterior probabilities. *Drawidajaponica* and *Pontoscolexcorethus* were included as outgroups.

**Figure 4.**
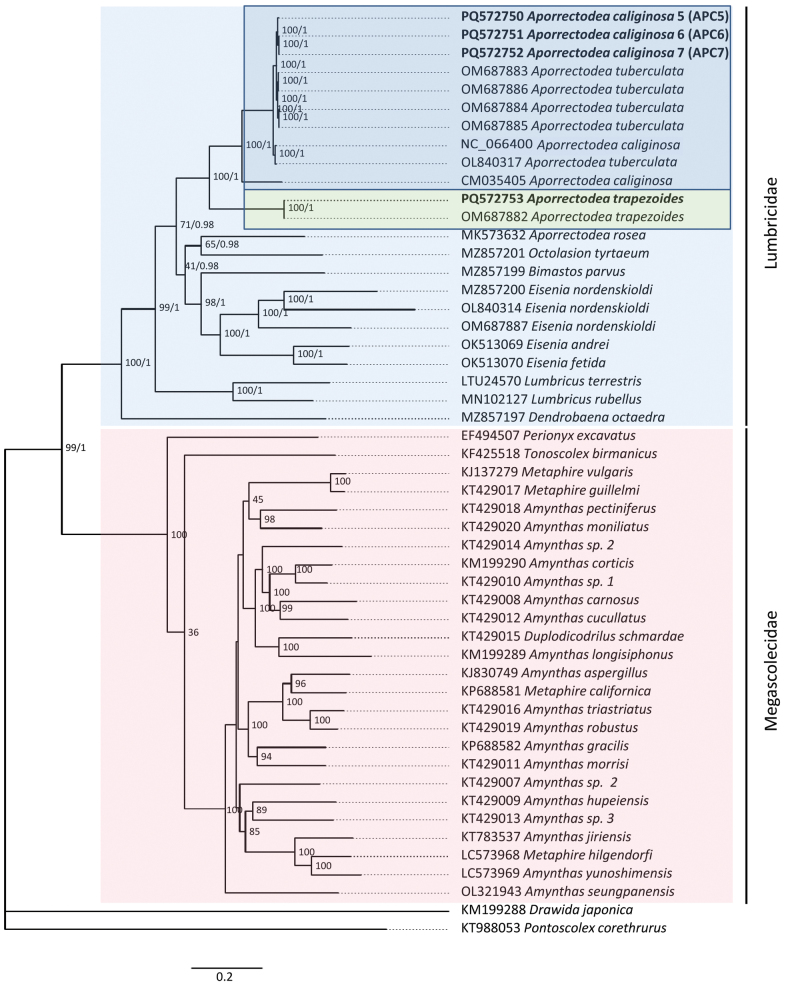
Phylogenetic analysis of 40 Megadrili group spp. using 13 PCGs, including *Aporrectodeatrapezoides* and *Ap.caliginosa*. Numbers beside nodes are ML bootstrap values and BI posterior probabilities. *Drawidajaponica* and *Pontoscolexcorethus* were included as outgroups.

**Figure 5.**
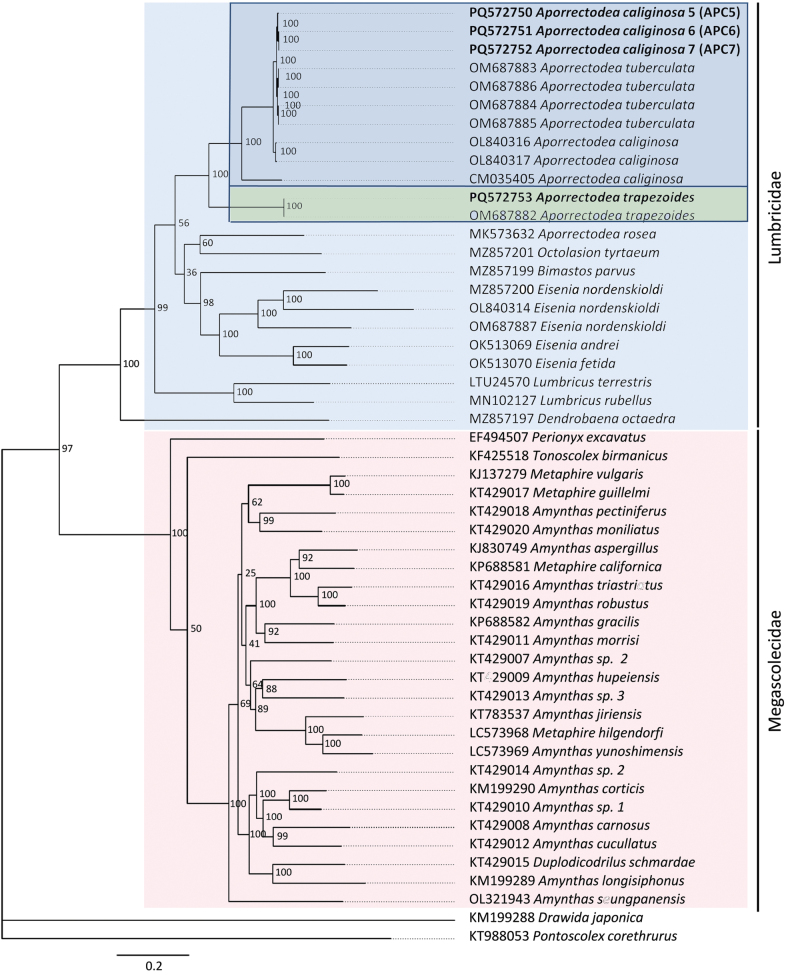
Partitioned phylogenetic analysis of 40 Megadrili group spp. using 13 PCGs, including *Aporrectodeatrapezoides* and *Ap.caliginosa*. Numbers beside nodes are ML bootstrap values.

Phylogenetic reconstruction of the available complete or nearly complete lumbricid mitogenomes and use of the 13 PCGs strongly supported the family Lumbricidae (100% bootstrap support). In addition, the genera *Eisenia* and *Lumbricus* were resolved as monophyletic, and the close relationship between *Ap.trapezoides/caliginosa* species pairs was confirmed (Figs 3–5). Consequently, the genus *Aporrectodea* was found to be polyphyletic, as *Ap.rosea* did not form a clade with the other analyzed *Aporrectodea* species.

The uncorrected *p*-distances of the mitogenomes (excluding the non-coding region) between *Ap.caliginosa/tuberculata* and *Ap.trapezoides* ranged from 16.1% to 16.4% (Table 5). The two *Ap.trapezoides* (from China and Korea) did not exhibit any genetic distance (0.00%).

**Table 5.** Genetic *p*-distances of selected Lumbricidae mitogenomes using the complete mitogenome sequences excluding the non-coding region.

*Ap.rosea* MK573632												
*Ap.trapezoides* OM687882	0.203											
*Ap.trapezoides* this study	0.203	0.000										
*Ap.caliginosa* CM035405	0.202	0.164	0.163									
*Ap.caliginosa* NC_066400	0.201	0.161	0.161	0.110								
*Ap.caliginosa* (Apc-5)	0.200	0.164	0.164	0.111	0.021							
*Ap.caliginosa* (Apc-6)	0.201	0.164	0.164	0.110	0.020	0.004						
*Ap.caliginosa* (Apc-7)	0.201	0.164	0.164	0.110	0.020	0.004	0.000					
*Ap.tuberculata* OL840317	0.200	0.161	0.161	0.109	0.006	0.021	0.020	0.020				
*Ap.tuberculata* OM687884	0.201	0.163	0.163	0.109	0.020	0.011	0.010	0.010	0.020			
*Ap.tuberculata* OM687885	0.201	0.163	0.163	0.109	0.020	0.011	0.010	0.010	0.020	0.000		
*Ap.tuberculata* OM687883	0.201	0.163	0.163	0.109	0.020	0.010	0.009	0.009	0.020	0.005	0.005	
*Ap.tuberculata* OM687886	0.201	0.163	0.163	0.109	0.020	0.010	0.009	0.009	0.020	0.005	0.005	0.000

The maximum genetic distance within *Ap.caliginosa* was 11.1% between a Hungarian specimen (Apc-5) and CM035405 (Azores Island, Portugal); however, between the L2 specimens identified as *Ap.tuberculata* and *Ap.caliginosa* it was only 2.1% (between Apc-5 and OL840317).

## ﻿Discussion

Here, we report the first complete mitogenomes of *Ap.caliginosa* and *Ap.trapezoides*. The newly analyzed mt genomes showed an arrangement and base composition typical of earthworms (Shekhovtsov et al. 2020; Csuzdi et al. 2022) and fit into the size distribution of previously known complete earthworm mitogenomes (14,648–16,560) (Csuzdi et al. 2022; Zhao et al. 2022). Our data are in agreement with the published incomplete *Ap.caliginosa*/*tuberculata* mitogenomes (15,058–15,129 vs 15,090–15,123) which demonstrates that the data from Zhao et al. (2022) are nearly complete, and only a few bp may be missing for circularity. For *Ap.trapezoides* (14,998 vs 15,014), this difference was also negligible.

Amino acid and base pair compositions across the 13 PCGs in *Ap.caliginosa* L2 and *Ap.tuberculata* species pairs were highly similar, showing 96–100% similarity across different genes. We obtained slightly lower similarity data when we compared *Ap.caliginosa* L2, and the sequence belonged to L3 (CM035405), indicating that the *Ap.tuberculata* sequences reported by Zhao et al. (2022) belong to *Ap.caliginosa*. This was clearly depicted in the phylogenetic reconstructions using the *cox1*, 13 PCGs, and complete mt genomes (Figs 1, 3, 4), corroborating the earlier findings of Briard et al. (2012) and Fernández et al. (2012) that *Ap.tuberculata* auct. (Eisen, 1874) is a synonym for *Ap.caliginosa* (Savigny, 1826). However, this was not observed for *Ap.trapezoides*, corroborating with previous results (Pérez-Losada et al. 2009; Fernández et al. 2011, 2012) and suggesting that *Ap.trapezoides* is distant from *Ap.caliginosa*, as it showed only 72.33%–91.56% nucleotide sequence similarity and 86.32%–99.4% amino acid composition similarity (lowest for *nad3* and highest for *cox1*; Tables 3, 4). This result was also reflected in the phylogenetic trees (Figs 1, 3, 4).

Phylogenetic analysis of the complete mt genomes (excluding the control region) and 13 PCGs strongly supported the monophyletic family Lumbricidae; however, the genus *Aporrectodea* proved to be polyphyletic. The species *Ap.rosea* does not form a clade with *Ap.caliginosa*/*trapezoides* sequences, reinforcing findings of previous studies’ (Domínguez et al. 2015; Marchán et al. 2022, 2023), and restricting *Aporrectodea* Örley, 1885 to the *Ap.caliginosa*/*trapezoides* complex and other closely related Franco-Iberian species (Marchán et al. 2023).

## ﻿Additional information

### Author ORCIDs

Csaba Csuzdi https://orcid.org/0000-0002-0319-7836

Jachoon Koo https://orcid.org/0000-0002-3559-326X

Yong Hong https://orcid.org/0000-0002-8093-9717

### Data availability

The genome sequence data supporting the results of this study are openly available in GenBank of NCBI under the accession no. PQ572750–PQ572753.
